# Plasma‐Activated Solutions Mitigates DSS‐Induced Colitis via Restoring Redox Homeostasis and Reversing Microbiota Dysbiosis

**DOI:** 10.1002/advs.202304044

**Published:** 2023-10-23

**Authors:** Tuanhe Sun, Kaijie Ren, Guimin Xu, Rulan Ma, Xueni Wang, Tianhao Min, Xin Xie, Anbang Sun, Yuyi Ma, Haonan Wang, Yong Zhang, Kun Zhu, Chengxue Dang, Guanjun Zhang, Hao Zhang

**Affiliations:** ^1^ Department of Surgical Oncology The First Affiliated Hospital of Xi'an Jiaotong University Xi'an Shaanxi 710061 China; ^2^ State Key Laboratory of Electrical Insulation and Power Equipment School of Electrical Engineering Xi'an Jiaotong University Xi'an Shaanxi 710061 China; ^3^ Department of Hepatobiliary Surgery The First Affiliated Hospital of Xi'an Jiaotong University Xi'an Shaanxi 710061 China; ^4^ Department of Nuclear Medicine The First Affiliated Hospital of Xi'an Jiaotong University Xi'an Shaanxi 710061 China

**Keywords:** microbiota, plasma activated solutions, redox homeostasis, ulcerative colitis

## Abstract

Ulcerative colitis is a chronic disease that increases the risk of developing colorectal cancer. Conventional medications are limited by drug delivery and a weak capacity to modulate the inflammatory microenvironment. Further, gut microbiota dysbiosis caused by mucosal damage and dysregulated redox homeostasis leads to frequent recurrence. Therefore, promoting mucosal healing and restoring redox homeostasis is considered the initial step in treating ulcerative colitis. Plasma‐activated solutions (PAS) are liquids rich in various reactive nitrogen species (RNS) and reactive oxygen species (ROS) and are used to treat multiple diseases. However, its effect on ulcerative colitis remains to be examined. Therefore, using a DSS‐induced mice colitis model, it is found that PAS has the potential to treat colitis and prevent its recurrence by promoting intestinal mucosal repair, reducing inflammation, improving redox homeostasis, and reversing gut microbiota dysbiosis. Further, an equipment is designed for preparing PAS without using nitrogen; however, after treatment with the Nitro‐free PAS, the therapeutic effect of PAS is significantly weakened or even lost, indicating that RNS may be the main mediator by which PAS exerts its therapeutic effects. Overall, this study demonstrates the treatment of ulcerative colitis as a novel application of PAS.

## Graphical abstract

Ulcerative colitis is a chronic recurrent intestinal disease with potential risk of colorectal carcinogenesis and bringing a heavy economic burden to the world. In this study, plasma‐activated solutions is found that is proven useful in various diseases have potential to promote intestinal mucosal repairing through multiple mechanism in colitis mouse model. In the future, this study will continue to conduct this study and attempt to evaluate its safety and effectiveness in human, providing new insights for the treatment and prevention of colitis.

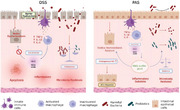

## 1 Introduction

Ulcerative colitis (UC) is a chronic, recurrent disease associated with abdominal cramps, bloody diarrhea, and mucinous stools. UC usually damages the colon and rectum, and this damage usually expands to the proximal areas of the colon.^[^ ^1^, ^2^ ^]^ The development of UC is a complicated process involving interactions between environmental, bacterial, and immune factors.^[^ ^3^ ^]^ Patients with UC present enhanced mucosal immune responses and bacterial dysbiosis.^[^ ^4^ ^]^ UC treatment involves oral or rectal therapies or surgery, depending on the activity, severity, and extent of inflammation and prognostic factors.^[^ ^1^ ^]^ Drugs currently used for treating UC include corticosteroids, 5‐aminosalicylic acid, immunomodulators, and biological agents. The aim of these treatments is to maintain remission^[^ ^5^ ^]^; however, strategies to promote mucosal healing and prevent recurrence remain lacking. More importantly, patients with UC usually have a high risk of developing colorectal cancer,^[^ ^1^ ^]^ which further challenges patient management.

Nitric oxide (NO), which commonly exhibits elevated levels in the colonic lumen of patients with UC, is now considered a harmful factor in colonic epithelial cells.^[^ ^6^ ^]^ Excessive NO impairs the metabolism of colonic epithelial cells, resulting in breakdown of the mucosal barrier and active UC. Bacterial dysbiosis may be an initiating factor in this process, leading to an excess of nitric oxide,^[^ ^7^ ^]^ as well as hydrogen sulfide, both of which disrupt the epithelial barrier. Bacteria then enter the lamina propria and stimulate immune cells, which activate the production of cytokines and nitric oxide to further aggravate damage.^[^ ^4^ ^]^ However, high NO concentrations may also protect immune cells by enhancing their ability to target bacteria.^[^ ^8^ ^]^ Therefore, balancing nitrate metabolism is vital for maintaining the colonic barrier. Cold atmospheric plasma (CAP), a fourth‐state matter produced by an electrical discharge process, is widely used in medical research because its temperature is close to room temperature.^[^ ^9^ ^]^ Plasma generated by ionization in air is rich in reactive nitrogen species (RNS), reactive oxygen species (ROS), and other high‐energy substances; further, it contains various short‐term and long‐term components, including hydroxyl radicals (OH), superoxide radicals (O^2−^), ozone (O_3_), atomic oxygen, singlet δ oxygen, peroxynitrite (ONOO), nitrogen dioxide radical (NO_2_^−^), and nitric oxide (NO); it can also participate in various biological processes.^[^ ^10^ ^]^ Preliminary studies have suggested that CAP exerts antitumor effects.^[^ ^11^, ^12^, ^13^, ^14^ ^]^ As ROS and RNS are also involved in signal transduction in biological processes, they tend to play a role in other processes associated with inflammatory diseases, such as regulating cell viability, proliferation, and inflammatory responses to promote wound healing.^[^ ^15^, ^16^, ^17^, ^18^ ^]^ Production of plasma‐activated solutions (PAS) involves the treatment of solutions with CAP, which is rich in long‐lived ROS and RNS. PAS is stable at room temperature; further, it has good fluidity, high safety, and great potential for clinical use.^[^ ^19^ ^]^

Considering the importance of the gut microbiota in UC, we hypothesized that PAS might contribute to the treatment of UC by regulating the gut microbiota and influencing its respiration in nitrate disorders. In this study, we first tested and standardized the components of PAS and verified their ability to treat UC and prevent remission. Next, we discussed the role of the gut microbiota during this process. We further developed a novel method to create nitro‐free PAS by discharging nitrogen into the environment, and examined the specific role of PAS in adjusting the respiration of nitrate in the gut microbiota.

## 2 Results

### 2.1 Experimental Setup and Preparation of Plasma‐Activated Solutions

**Figure 1:**
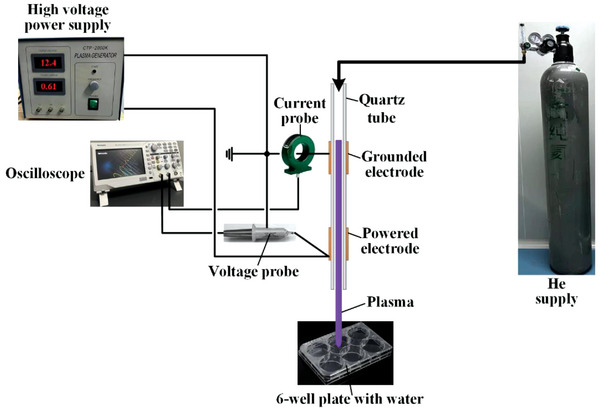
Experimental setup and preparation of the plasma‐activated solution.

The plasma device used in this study was the same as described previously.^[^ ^20^, ^21^ ^]^ It comprised a quartz tube with an inner radius of 1 mm and an outer radius of 2 mm (**Figure** **1**). Two 1 cm wide copper strips were wrapped outside the tube as grounded and powered electrodes. The flow rate of the helium working gas (99.9999%) in the capillary was set to 15 standard L min^−1^ using a flow controller. As shown in Figure 1, the electrodes were connected to a resonant high‐voltage power supply (CTP‐2000K, Suman Electronics Co., Ltd.), and the applied voltage between the two outer electrodes was measured using a high‐voltage probe (P6015A, Tektronix Inc.). The current passing through the grounded electrode was measured using a current probe (Pearson 2877, Pearson Electronic Inc.). The two signals were recorded using a digital oscilloscope (TBS1102C, Tektronix, Inc.). In this study, a gas discharge plasma was generated under a sinusoidal wave format of 20 kHz with a peak voltage of 12.4 kV.

For preparing PAS, 6 mL of normal water was first added to each well of a 6‐well plate and then treated with plasma for 0 min (control), 30 s, 1, 2, 10, and 30 min. The distance between the end of the dielectric plasma tube and the bottom of the 6‐well plate was ≈3 cm.

### 2.2 PAS Administered for 2 min has Therapeutic Potential for DSS‐Induced Colitis

**Figure 2:**
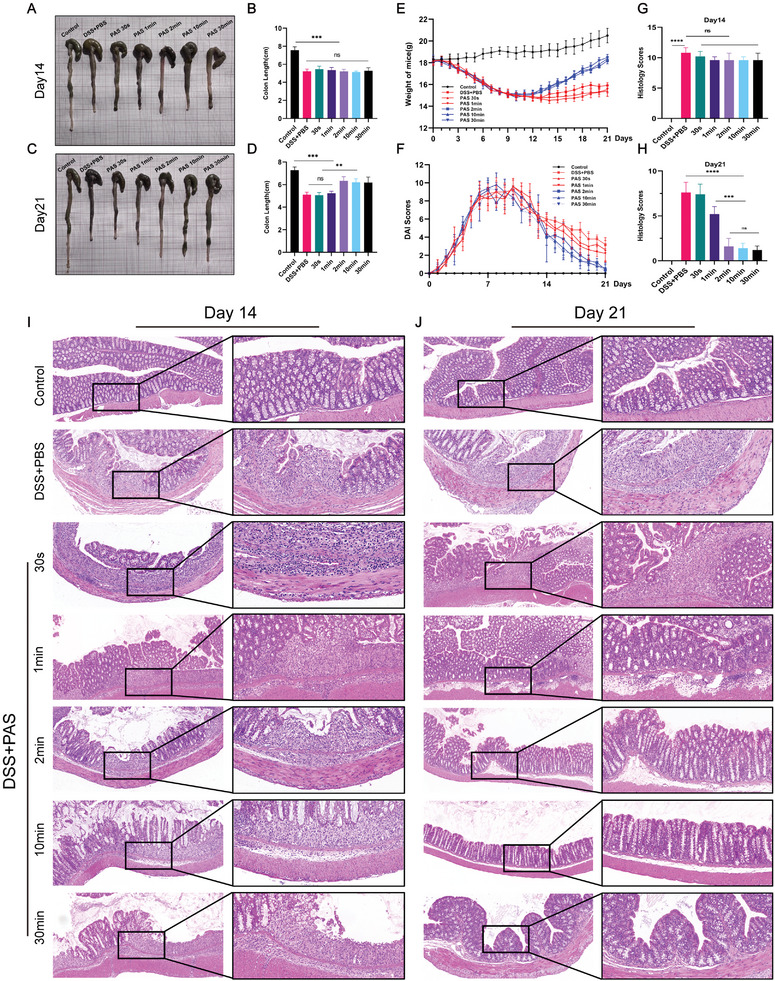
PAS 2 min has therapeutic potential for DSS‐induced colitis. A) Macroscopic images and B) lengths of colons in each group on day 14. On day 14, the colon lengths of mice in the PBS or PAS‐treated groups were shorter than those in the control group. C) Macroscopic images and D) colon lengths of each group on day 21. On day 21, the colon lengths of mice in PBS‐treated, PAS 30 s, and PAS 1 min groups were shorter than those in the control group, whereas the colon lengths of the PAS 2 min, PAS 10 min, and PAS 30 min groups were slightly shorter than those in the control group. E) Daily body weight changes following treatment. The weight of mice in the PAS 2 min, PAS 10 min, and PAS 30 min groups was recovered to that before the DSS intervention; however, the weight of mice in the PBS‐treated, PAS 30 s, and PAS 1 min groups was still lower than that in the control group. F) Disease activity index (DAI) scores following treatment. The DAI scores of mice in the PAS 2 min, PAS 10 min, and PAS 30 min groups were slightly higher than those in the control group, whereas the DAI scores of mice in the PBS‐treated, PAS 30s, and PAS 1 min groups were significantly higher than those in the control. G) Histological scores of colons on day 14. On day14, the histological scores in the PBS‐ and PAS‐treated groups were higher than those in the control group. H) Histological scores of colons on day 21. On day 21, the histological scores in the PBS‐treated, PAS 30 s, and PAS 1 min groups were higher than those in the control group, while those in the PAS 2 min, PAS 10 min, and PAS 30 min groups were lower than those of the above three groups but slightly higher than those in the control; I) H&E‐stained colon sections on day 14. On day 14, H&E staining of both PBS‐treated and PAS‐treated groups still showed obvious colonic injury. J) On day 21, H&E staining of PBS‐treated, PAS 30s, and PAS 1 min groups still showed obvious colonic injury, while that of the PAS 2 min, PAS 10 min, and PAS 30 min groups was almost normal, with only a small amount of inflammatory cell infiltration remaining. Data were presented as Mean ± SD and comparisons were performed with Student's *t*‐test (B, D, G, H) or two‐way ANOVA with Tukey's test (E, F). Sample size (n) for each statistical analysis was 5. ^**^ *p* < 0.01, ^***^ *p* < 0.001, ^****^ *p* < 0.0001, ns: no significance.

To evaluate the therapeutic potential of PAS with different processing times in a mouse model of colitis, mice were treated with PAS for the abovementioned treatment durations. The results showed that on day 14, the colon lengths of PAS‐treated mice had no significant difference compared to the PBS‐treated group, and both were obviously shorter than that of the control group (**Figure** **2**A,B). On day 14, the body weights of the PAS 2, 10, and 30 min groups were higher than those of the PBS‐treated, PAS 30 s and 1 min groups, but there was no significant difference between them (Figure 2E). Compared to the PBS group, there was no decrease in the disease activity index (DAI) in each PAS group on day 14 (Figure 2F). Hematoxylin‐eosin (H&E) staining revealed no significant difference in the degree of colonic injury between the PAS and PBS groups on day 14 (Figure 2I). Each group exhibited severe mucosal destruction, submucosal edema, and extensive inflammatory cell infiltration, with no significant difference in histological scores (Figure 2G). Therefore, the use of PAS for 1 week did not alleviate dextran sulphate sodium (DSS)‐induced colitis. Therefore, we extended the experimental period and continued with our observations. On day 21, after 2 weeks of PAS treatment, the colons of mice in the PBS‐treated, PAS 30s and PAS 1 min groups were significantly shorter than those of the normal control group (Figure 2C,D), whereas the colons of the PAS 2 min, PAS 10 min, and PAS 30 min groups returned to normal lengths but were still slightly shorter than those of the normal control mice. Regarding body weight, the mice in the PAS 2 min, PAS 10 min, and PAS 30 min groups all recovered their body weights to that before the DSS intervention (Figure 2E). The mice in the PAS 2 min, PAS 10 min, and PAS 30 min groups showed weight loss but no symptoms such as diarrhea or fecal blood (Figure 2F). H&E staining of the PAS 2 min, PAS 10 min, and PAS 30 min groups suggested that only a small amount of inflammatory cell infiltration remained in the colonic mucosa of mice, which was recovered to the level before DSS intervention, whereas the PBS‐treated, PAS 30 s, and PAS 1 min groups still showed obvious colonic injury (Figure 2J). The histological scores of the PAS 2 min, PAS 10 min, and PAS 30 min groups returned to normal, whereas those of the other groups remained high (Figure 2H). Based on these experimental results, and considering the time and preparation cost, PAS 2 min showed both therapeutic potential and convenience; therefore, we used PAS 2 min as the standard treated condition in subsequent experiments.

### 2.3 Nitrogen in PAS may be the Main Component Contributing to the Therapeutic Effect

**Figure 3:**
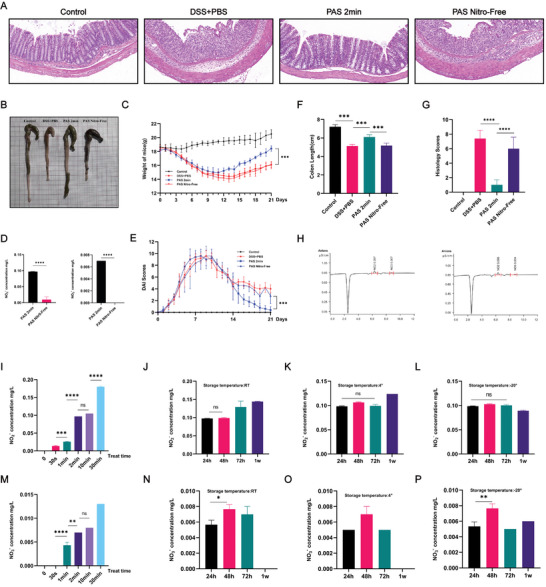
Nitrogen in PAS may be the main component contributing to its therapeutic effect. A) H&E‐stained colon sections. H&E staining showed obvious colonic inflammation and injury after the Nitro‐free PAS treatment. B) Macroscopic images of colons and F) the lengths of colon in each group. After treatment with PAS for two weeks, the colons in the PAS 2 min group were longer than those in the PBS‐treated group. However, after treatment with Nitro‐free PAS, the therapeutic effect disappeared and the colons in Nitro‐free PAS were shorter than those in the PAS 2 min group. C) Daily body weight changes following treatment. After treatment with Nitro‐free PAS, the recovery of weight in the PAS 2 min group disappeared. The weight of mice in the Nitro‐free PAS group was significantly reduced compared to that in the control group; D) Concentrations of nitrate and nitrite in PAS 2 min and Nitro‐Free PAS measured using ion chromatography. Nitrite concentrations in Nitro‐free PAS were significantly lower than those in the PAS 2 min group while nitrite could not even be detected in the Nitro‐Free PAS. E) DAI scores following treatment. The DAI scores of the Nitro‐Free PAS group were significantly higher than those in the control and PAS 2 min groups. G) Histological scores of colons. On day 21, H&E staining of Nitro‐Free PAS groups still showed obvious colonic injury, whereas H&E staining of the PAS 2 min group almost returned to normal. H) Representative graph of nitrate and nitrite concentrations measured using ion chromatography. I) The concentration of nitrite under different treatment durations. The overall trend was that the concentration of nitrite increased gradually with the extension of treatment time. The concentration of nitrite in the PAS 30 min group was significantly higher than that in the other groups. No statistically significant difference was found between the PAS 2 min and 10 min groups, but the levels in both were higher than those in the shorter PAS treatment. J–L) The concentration of nitrite under different storage conditions. The concentration of nitrite in PAS was stable at room temperature within 24 h. M) The concentration of nitrate under different treatment durations. The concentration of nitrite increased gradually with the extension of treatment time. No statistically significant difference was found between PAS 2 and 10 min, but the concentrations in both were higher than those in the shorter PAS treatments. N–P) The concentration of nitrate under different storage conditions. At room temperature, the concentration of nitrate remained stable within 24 h. Data were presented as Mean ± SD and comparisons were performed with Student's *t*‐test (D, F, G, I‐P) or two‐way ANOVA with Tukey's test (C, E). Sample size (n) for each statistical analysis was 5. ^**^ *p* < 0.01, ^***^ *p* < 0.001, ^****^ *p* < 0.0001, ns: no significance.

PAS is a mixture of various ROS/RNS. As superoxide anions, free radicals, singlet oxygen, and other particles exist for a very short time, PAS is eventually converted to hydrogen peroxide and nitrate/nitrite; detection based on hydrogen peroxide and nitrate/nitrite is currently the most used and convenient detection method for PAS components. Previous studies have shown that ROS often plays an initiating and aggravating role in colitis development, and some studies have found that exogenous administration of nitrate can effectively alleviate DSS‐induced colitis. Therefore, we speculated that the therapeutic effect of PAS may be attributed to RNS. Based on the above assumptions, we developed Nitro‐free PAS generation equipment for preparing nitrogen oxide‐free PAS and verified that the nitrate/nitrite levels decreased or even disappeared through ion chromatography (**Figure** **3**D). The results showed that after two weeks of nitro‐free PAS treatment, the therapeutic effect of PAS was significantly weakened or even lost. The colon length of these mice was shorter than that of the control group and PAS 2 min group (Figure 3B,F). Further, the body weights of these mice were significantly lower than those of the mice in the other two groups (Figure 3C), and the DAI was significantly higher (Figure 3E). H&E staining showed obvious colonic inflammation and injury after nitro‐free PAS treatment, and the histological score was significantly higher than that of the above two groups (Figure 3A,G). Therefore, we believe that the RNS may be the main mediator by which PAS exerts its therapeutic effects.

To standardize PAS production, ion chromatography was used to measure the nitrate/nitrite concentration in PAS at different processing times and in the PAS 2 min group under different storage conditions. The results showed that the concentration of nitrate/nitrite in PAS increased with prolonged treatment time (Figure 3I,M). There was a slight difference between the 2 and 10 min treatment groups, and the level of nitrate/nitrite was significantly higher than that in the above two groups after 30 min of treatment. Considering the food safety and existing animal experimental results, using PAS 2 min in mice with DSS‐induced colitis is effective, convenient, and safe. We further explored the optimal storage conditions for PAS. The ion chromatography results showed that the nitrite and nitrate contents remained stable for 24 h at room temperature. After 48 h, the nitrate content fluctuated significantly, and after 72 h, the nitrite content increased; however, nitrate could not be detected at 1 week (Figure 3N). Therefore, it was appropriate to use PAS 2 min within 24 h at room temperature. Under storage at 4 °C, nitrite was stable for 72 h, but there was a significant increase in its levels after 1 week, and nitrate was only stable for 48 h. Nitrite was stable for one week under storage conditions of −20 °C, whereas nitrate content still fluctuated obviously after 48 h. Based on these results, it should be used within 48 h after PAS preparation.

### 2.4 PAS 2 min Alleviated Inflammation, Regulated Redox Homeostasis, and Promoted Mucosal Repair

**Figure 4:**
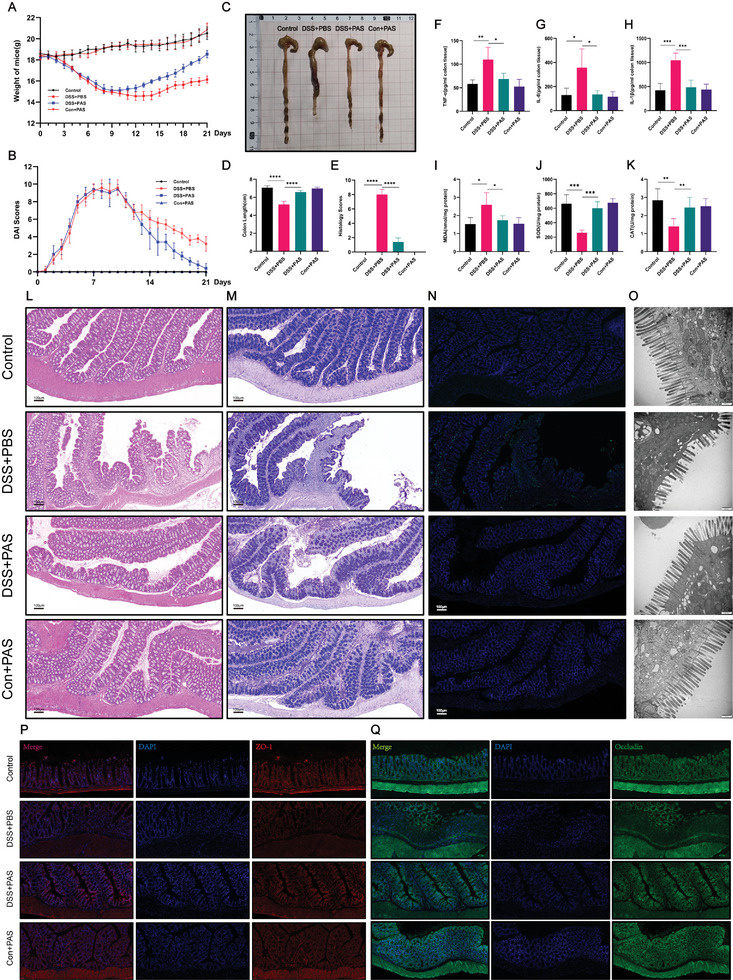
The PAS 2 min treatment alleviated inflammation, regulated redox homeostasis, and promoted mucosal repairing. A) Daily body weight changes following treatment. After PAS 2 min treatment for two weeks, the weight of the mice returned to normal and was significantly higher than that of the PBS‐treated animals. Further, there was no statistically significant difference in weight between the control and control+PAS groups. B) Disease activity index (DAI) scores following treatment. The DAI scores of mice returned to normal levels after 2 weeks of PAS 2 min treatment, whereas the scores of the PBS group remained at a high level. C) Macroscopic images of the colons and D) colon lengths. The colon lengths of mice in the PBS‐treated groups were still shorter than those in the control group, whereas those in the PAS 2 min group were only slightly shorter than those in the control group. E) Histological scores of colons. Histological scores of colons in the PBS‐treated group were higher than those in the control group, whereas those in the PAS 2 min group were lower than those in the PBS‐treated group but slightly higher than those in the control. F–H) The concentrations of TNF‐α, IL‐6, and IL‐1β in colon tissue. The increased concentrations of TNF‐α, IL‐6, and IL‐1β in colon tissue induced by DSS could be reversed by the PAS 2 min treatment for 2 weeks. I) MDA concentrations in colon tissue. The concentration of MDA was upregulated in the DSS+PBS group compared with that in the PAS 2 min and control groups. J, K) Concentrations of SOD/CAT in the colon tissue. The concentrations of SOD/CAT were decreased in the DSS+PBS group compared with those in the PAS 2 min and control group. N) Representative fluorescent images of TUNEL staining in the colonic sections. Scale bars represent 100 µm. O) Representative transmission electron microscopy (TEM) images for the microstructure of colonic epithelia. P) Expression of ZO‐1 determined using confocal immunofluorescence. Q) Expression of occludin determined using confocal immunofluorescence. Data were presented as Mean ± SD and comparisons were performed with Student's *t*‐test (D‐K) or two‐way ANOVA with Tukey's test (A, B). Sample size (n) for each statistical analysis was 5. ^**^ *p* < 0.01, ^***^ *p* < 0.001, ^****^ *p* < 0.0001, ns: no significance.

After treatment with PAS 2 min, we further investigated the mechanism of action for PAS in colitis treatment. First, consistent with previous results, the colon length, body weight, and DAI of mice returned to normal levels after 2 weeks of PAS treatment (**Figure** **4**A–C). Compared with the PBS group, the serum levels of inflammatory factors IL‐6, TNF‐α, and IL‐1β in the PAS 2 min group were significantly lower, indicating that PAS could reverse the colonic inflammation induced by DSS (Figure 4F–H). We also measured the levels of oxidative stress indicators in the colon, and found that the levels of malondialdehyde (MDA), superoxide dismutase (SOD), and catalase (CAT) almost returned to normal, showing significant differences compared with those in the PBS group (Figure 4I–K). Next, we demonstrated the therapeutic effects of PAS at the histological level. The results of H&E and Alcian blue‐periodic acid‐schiff (AB‐PAS) staining suggested that intestinal epithelial damage in the mice was almost repaired after 2 weeks of treatment with PAS 2 min (Figure 4L). The main manifestations observed using H&E staining included obvious repair of mucosal damage, significantly reduced inflammatory cell infiltration in the mucosa, and almost complete disappearance of inflammatory cell infiltration in the submucosa; however, mild edema of the submucosa persisted. AB‐PAS staining indicated that the secretory function of the intestinal cells was recovered well (Figure 4M). Further, TdT‐mediated dUTP nick‐end labeling (TUNEL) staining showed that treatment of PAS 2 min reversed DSS‐induced colonic epithelial cell apoptosis (Figure 4N). Transmission electron microscopy (TEM) observation also showed that the loss of microvilli in colonic epithelial cells induced by DSS was reversed by treatment of PAS 2 min (Figure 4O). Previous studies have shown that ZO‐1 and Occludin are important markers for the integrity of the colonic epithelial barrier and the promotion of colonic epithelial cell damage repair. Therefore, we performed immunofluorescence (IF) for these tissues and found that DSS reduced the levels of ZO‐1 and occludin in the colon. After treatment of PAS 2 min, the expression level of these two proteins was increased to promote the remodeling of the intestinal epithelial mucosal barrier (Figure 4P,Q).The expression of ZO‐1 and Occludin in colons in each group at protein and mRNA level were analyzed by western blot and qRT‐PCR (Figure S1, Supporting Information). Further, to verify whether PAS had any adverse effects on the normal intestinal tract, we established a Control+PAS group at each step of the experiment and found that PAS had no adverse effects on the normal intestinal tract. In summary, treatment of PAS 2 min inhibited DSS‐induced colonic inflammation, regulated redox homeostasis, and promoted colonic mucosal epithelial repair.

### 2.5 Microbiota Transplantation from Mice Treated with PAS 2 min Relieves Colitis Better than PAS‐Derived Sterile Fecal Filtrate

**Figure 5:**
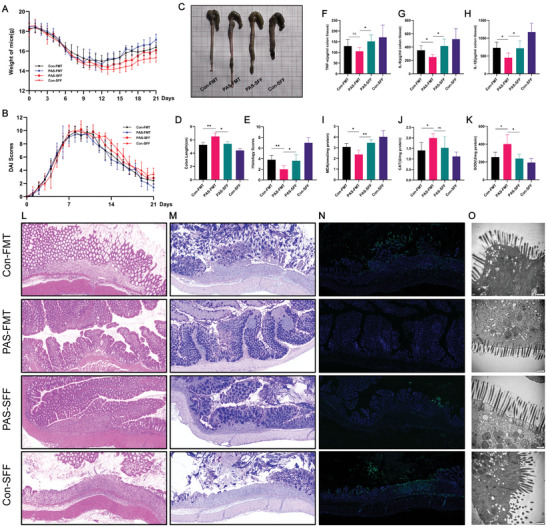
PAS microbiota transplantation relieves colitis better than the PAS‐derived sterile filtrate. A) Daily body weight changes following treatment. The weight of mice in the PAS‐FMT group was significantly higher than the that in the PAS‐SFF, CON‐FMT, and CON‐SFF groups. B) Disease activity index (DAI) scores following treatment. DAI scores in the PAS‐FMT group were significantly lower than those in the PAS‐SFF, CON‐FMT, and CON‐SFF groups. C) Macroscopic images of colons. The mice in the PAS‐FMT group had the least severe colon damage and inflammation. D) Colon lengths. The colons of mice in the PAS‐FMT group were significantly longer than those in the PAS‐SFF, CON‐FMT, and CON‐SFF groups. E) Histological scores of colons. The histological scores of colons in the PAS‐FMT group were the lowest among the four groups. F–H) Concentrations of pro‐inflammatory cytokines. I) MDA concentrations in colon tissue. The concentration of MDA in the PAS‐FMT group was lower than that in the other three groups. J) Concentrations of CAT in colon tissue. The concentrations of CAT in the PAS‐FMT and PAS‐SFF groups were lower than those in the other two groups, but the difference between the PAS‐FMT and PAS‐SFF groups was not statistically significant. K) SOD concentrations in colon tissue. The concentration of SOD in the PAS‐FMT group was lower than that in the other three groups. L) H&E‐stained colon sections. Colon injury was the mildest in mice of the PAS‐FMT group among the four groups. M) Alcian blue‐periodic acid‐Schiff (AB‐PAS)‐stained colon sections. N) Representative fluorescence images of TUNEL staining in the colonic sections. Scale bars represent 100 µm; O) Representative transmission electron microscopy (TEM) images for the microstructure of colonic epithelia. Data were presented as Mean ± SD and comparisons were performed with Student's *t*‐test (D–K) or two‐way ANOVA with Tukey's test (A, B). Sample size (n) for each statistical analysis was 5. ^**^ *p* < 0.01, ^***^ *p* < 0.001, ^****^ *p* < 0.0001, ns: no significance.

Because most gut microbiota are obligate or facultative anaerobic bacteria, they survive in nitrogen and its compounds under hypoxic conditions. We found that reactive nitrogen is an essential component of PAS in the treatment of colitis. To further explore the potential mechanism of PAS in the treatment of colitis and to determine whether it can treat colitis by affecting the gut microbiota, we designed another experiment. The results showed that the body weight of mice in the CON‐FMT (fecal microbiota transplantation) group changed starting on day 11 and increased slowly, whereas the weight of mice in the PAS‐FMT group began to recover on day 12 and was significantly higher than that in the other three groups after 2 weeks (**Figure** **5**A). Further, we found that the body weight of the CON‐FMT group was higher than that of the PAS‐SFF group, suggesting that the gut microbiota, rather than metabolites, has potential application value in treating colitis (Figure 5A). The DAI of the mice in the PAS‐FMT group decreased significantly after the intervention and was significantly lower than those in the other three groups (Figure 5A). However, unlike the body weights, the DAI of the PAS‐SFF (sterile fecal filtrate transplantation) group were lower than those of the CON‐FMT group, mainly because the number of positive cases for fecal occult blood in mice decreased and the shape of the feces changed. The colon lengths of the PAS‐FMT group were significantly longer than that of the other three groups, whereas there was no significant difference between the CON‐FMT and the PAS‐SFF or SFF groups (Figure 5C). The inflammatory factor levels showed that the serum IL‐1β, IL‐6, and TNF‐α levels in the PAS‐FMT group were lower than those in the other three groups (Figure 5F–H), and all differences were significant. Regarding tissue oxidative stress, the levels of CAT and SOD in the tissues increased significantly after PAS‐FMT treatment, whereas those in the other three groups were lower than those in the PAS‐FMT group (Figure 5J,K). Further, PAS‐FMT reduced the MDA levels in tissues (Figure 5I). These results indicate that PAS‐FMT reduced inflammation and improved oxidative stress. At the histological level (Figure 5L), mucosal repair in the PAS‐FMT group was significantly better than that in the other three groups. Although colonic damage was still observed, most of the damage was limited to the mucosal layer. Further, the muscularis mucosae were completely repaired and the edema of the submucosa disappeared. AB‐PAS staining also suggested that the mucus secretion function of the colonic mucosa was significantly better than that in the other three groups (Figure 5M). These results demonstrated that colitis treated by PAS partly by affecting the composition of the gut microbiota, rather than its metabolites. The TUNEL staining's results were consistent with the above observations, and the level of colonic apoptosis in the PAS‐FMT group was significantly lower than that in the other three groups (Figure 5N). The TEM observation also suggested that at the microscopic level, PAS‐FMT restored colonic microvilli, whereas the effects in the other three groups were far weaker than these (Figure 5O). Based on these findings, we believe that the therapeutic effect of PAS on colitis occurs partly via modulation of the gut microbiota. Therefore, we performed 16S rDNA sequencing of mice treated with PAS for colitis.

### 2.6 PAS 2 min can Reverse Microbiota Dysbiosis

**Figure 6:**
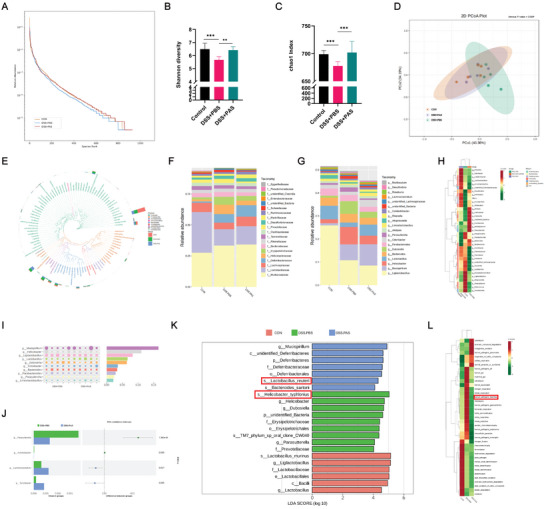
PAS 2 min treatment can restore intestinal bacterial homeostasis. A) Operational taxonomic unit (OTU)‐based group rank Abundance curve. The Rank Abundance Curve indicated that the richness and diversity of intestinal bacteria in the PBS intervention group were significantly lower than those in the control and PAS treatment groups. B) Shannon index and C) Chao1 index between the three groups. The Shannon and Chao1 indices were higher in the PAS intervention group than in the PBS intervention group. D) Weighted Unifrac distance principal coordinate analysis (PCoA) analysis based on OTUs. The bacterial composition of the control and PAS groups was similar, whereas that of the PBS group was significantly different. E) Phylogenetic relationships at the genus level based on OTUs. The Lactobacillus genus was increased after the PAS 2 min treatment. F,G) Bar chart of the relative abundance at the family and genus level of the top ten groups. H) Species notes and abundance information heat maps for each sample at the genus level. I) SIMPER (Similarity Percentage) analysis at the genus level. J) Difference between DSS+PBS and DSS+PAS at the genus level. PAS 2 min treatment increased the abundance of Lachnoclostridium. K) Analysis of differences in microbial taxa shown by Linear discriminant analysis Effect Size (LEfSe). At the species level, a significant enrichment in Helicobacter typhlonius was observed in the PBS treatment group, whereas enrichment in Lactobacillus reuteri could be seen at the species level in the PAS‐treated group; L) FAPROTAX functional prediction enrichment analysis based on OTUs. The significant enrichment in diarrhea pathways in the PBS group was reversed after PAS intervention.

Our previous experimental results revealed that the role of PAS in colitis treatment is partly achieved by regulating the gut microbiota; therefore, we performed 16S rDNA sequencing. The Rank Abundance Curve indicated that the richness and diversity of gut bacteria in the PBS intervention group were significantly lower than those in the control and PAS treatment groups (**Figure** **6**A). The Shannon index (Figure 6B) was significantly lower in the PBS intervention group than in the control group. However, the Shannon index was higher in the PAS intervention group than in the PBS intervention group, indicating that the PAS‐treated group had a higher diversity of gut microbiota. Further, the Chao1 index of the PAS intervention group was higher than that of the PBS intervention group (Figure 6C). These results indicate that PAS can restore the diversity of the gut microbiota, which may be an important mechanism underlying its function. Next, based on the weighted UniFrac distance, we conducted principal coordinate analysis (PCoA) to explore similarities in the bacterial composition of the three groups (Figure 6D). The results indicated that the bacterial composition of the control and PAS groups was similar, while that of the PBS group was significantly different (*P* = 0.019), indicating that after PAS intervention, the composition of the gut microbiota was similar to that of the healthy mice in the control group, further supporting our point. To further explore the specific microbes affected by PAS, we constructed a genus‐level phylogenetic tree based on the sequencing results. The results showed that the genus *Lactobacillus*, which is a common intestinal probiotic, was significantly decreased in the PBS group (Figure 6E). In addition to decreased levels in the microbiota of patients with colitis, *Lactobacillus* was recovered after PAS treatment, indicating that PAS has the potential to increase the levels of probiotics belonging to *Lactobacillus*. Further, the harmful bacterium *Helicobacter* in the PBS group showed significantly increased levels, and after intervention with PAS, the level of *Helicobacter* was reduced, indicating that PAS may also have the ability to regulate the number of harmful bacteria. Next, we selected the top ten species with the highest relative abundance at the family and genus levels in each group to draw a bar graph and obtained similar results; PAS treatment increased the abundance of the beneficial bacterial genus *Lactobacillus* and reduced the abundance of the harmful genus *Helicobacter*. Figure 6H shows a heatmap based on the operational taxonomic units (OTUs), demonstrating the enrichment of the harmful bacterium *Helicobacter* in the PBS treatment group, consistent with the above conclusion. To quantify the contribution of each species to the differences between the two groups, a SIMPER (Similarity Percentage) analysis was performed to reveal the top ten contributors at the genus level. Next, we searched for the genus with the most obvious differences and performed a *t*‐test. The results showed that PAS significantly decreased the increased levels of *Parasutterella* genus in the PBS group. *Parasutterella* has been proven as an opportunistic pathogen associated with obesity and type 2 diabetes. PAS also increased the *Lachnoclostridium* abundance (Figure 6J). The use of anti‐inflammatory drugs has been shown to increase the abundance of *Lachnoclostridium* to relieve colonic inflammation. At the species level, LEfSe (Linear discriminant analysis Effect Size) showed significant enrichment of *Helicobacter typhonius*, a proven harmful bacterium with definite pathogenicity to humans and animals, in the PBS treatment group, whereas enrichment in *Lactobacillus reuteri* was observed at the species level in the PAS‐treated group (Figure 6K). *Lactobacillus reuteri* has a strong ability to adhere to the intestinal mucosa, which can improve the distribution of the gut microbiota, antagonize the colonization of harmful bacteria, and prevent intestinal diseases. As PAS can increase the abundance of *Lactobacillus reuteri*, which may be one of the important reasons why PAS exerts its therapeutic potential through the gut microbiota. Finally, we performed FAPROTAX functional prediction enrichment analysis based on OTUs, and the results showed that significant enrichment of diarrhea pathways in the PBS group was reversed after PAS intervention, consistent with our previous observations in mice (Figure 6L). In summary, based on our speculation and the 16S rDNA sequencing results, PAS can improve the gut microbiota disorder induced by DSS, demonstrating the potential to reduce the levels of the harmful bacterium *Helicobacter typhonius* and increase the distribution of the beneficial bacterium *Lactobacillus reuteri*. These findings also verify that the regulation of the gut microbiota is one of the mechanisms by which a therapeutic effect is exerted.

### 2.7 PAS 2 min Pretreatment can Partly Prevent DSS‐Induced Mucosal Damage

**Figure 7:**
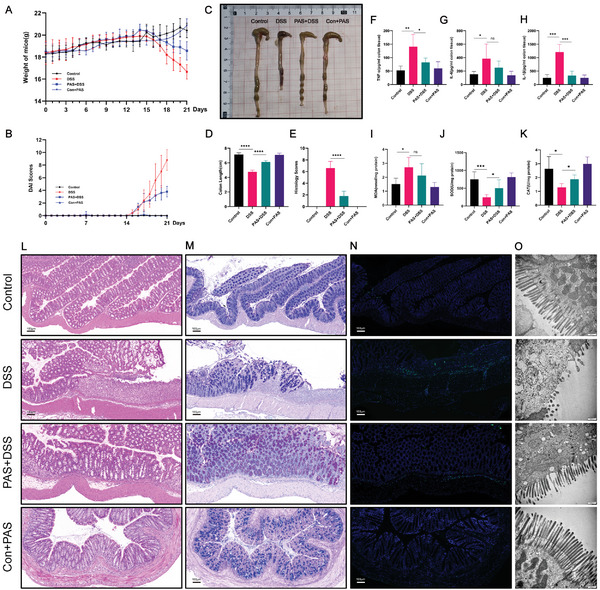
PAS 2 min pretreatment can partly prevent DSS‐induced mucosal damage. Daily body weight changes following treatment. After 2 weeks of PAS 2 min pre‐treatment, the weight loss of mice was not as obvious as that in the DSS group. B) Disease activity index (DAI) scores following treatment. The DAI scores in the PAS 2 min pre‐treatment group were lower than those in the DSS group. C) Macroscopic images of colons. PAS 2 min pre‐treatment can partly prevent DSS‐induced colon injury and inflammation. D) The lengths of colons in each group. The lengths of colons in the PAS 2 min pre‐treatment group were longer than those in the DSS group. E) Histological scores of colons. The histological scores of colons in the PAS 2 min pre‐treatment group were lower than those in the DSS group. F) The concentrations of TNF‐α in colon tissue. The concentration of TNF‐α was higher in the DSS group than that in the PAS 2 min pre‐treatment group. G) The concentrations of IL‐6 in colon tissue. There were no statistically significant differences between the PAS 2 min pre‐treatment and DSS groups, and both showed upregulation compared with that in the control group. H) The concentrations of IL‐1β in colon tissue. The concentration of IL‐1β was higher in the DSS group than in the PAS 2 min pre‐treatment group. I) The concentrations of MDA in colon tissue. There was no statistically significant difference between the PAS 2 min pre‐treatment and DSS groups, and both were upregulated than those in the control group; J) The concentrations of SOD and K) CAT in colon tissue. The concentrations of SOD/CAT were downregulated in both the DSS and PAS 2 min pre‐treatment groupd compared with those in the control group. L) H&E stained and M) Alcian blue‐periodic acid‐Schiff (AB‐PAS)‐stained colon sections. N) Representative fluorescence images of TUNEL staining in the colonic sections. Scale bars represent 100 µm. O) Representative transmission electron microscopic images (TEM) for colonic epithelial microstructure. Data were presented as Mean ± SD and comparisons were performed with Student's *t*‐test (D–K) or two‐way ANOVA with Tukey's test (A, B). Sample size (n) for each statistical analysis was 5. ^**^ *p* < 0.01, ^***^ *p* < 0.001, ^****^ *p* < 0.0001, ns: no significance.

Recurrence is a clinical characteristic of UC. After observing the therapeutic potential of PAS, we further explored its effect on recurrence, and the results showed that after 2 weeks of PAS 2 min pre‐treatment, the weight loss of the mice was not as obvious as that in the DSS group (**Figure** **7**A). DAI analysis also showed the same results (Figure 7B). Although the colon lengths of the PAS 2 min‐treated group were shorter than that of the control group, it was significantly longer than that of the direct DSS treatment group (Figure 7C). These results proved that PAS 2 min can prevent colitis recurrence. Further, the serum TNF‐α and IL‐1β levels in the PAS 2 min‐treated group were lower than those in the direct intervention group, but there was no significant difference in IL‐6 levels (Figure 7F–H). As for redox factors, the SOD and CAT levels in the PAS 2 min group were significantly decreased compared with that in the direct intervention group (Figure 7J,K), but the MDA level showed no difference (Figure 7I). H&E and AB‐PAS staining showed that the degree of colonic mucosal damage in the PAS 2 min pre‐treated group was significantly less severe than that in the DSS directly treated group (Figure 7L,M). The TUNEL staining indicated that the apoptosis level in the PAS 2 min group was lower than that in the DSS (Figure 7N). TEM observation also revealed less injury after PAS pre‐treatment (Figure 7O). Pre‐treated with PAS 2 min for 2 weeks and then DSS for one week, the expression of ZO‐1 and Occludin at protein and mRNA level in colons were upregulated compared with that pre‐treated with PBS (Figure S2, Supporting Information). These results suggest that pre‐treatment with PAS can reduce DSS‐induced colitis and that long‐term use of PAS has the potential to prevent colitis recurrence.

## 3 Discussion

The incidence of colitis is increasing annually, resulting in a serious economic burden,^[^ ^22^ ^]^ and its high recurrence rate remains a challenge for clinicians. Genetic susceptibility to colitis shows obvious familial aggregation.^[^ ^23^ ^]^ Environmental factors such as diet, smoking, lifestyle, and air pollution are also the main reasons for the rising incidence of colitis.^[^ ^24^ ^]^ Currently, two main types of 5‐aminosalicylic acid (5‐ASA)‐containing formulations, mesalamine/5‐ASA and sulfasalazine (SASP), are used as first‐line treatments for UC.^[^ ^25^ ^]^ However, owing to the poor compliance of some patients,^[^ ^26^ ^]^ a risk of recurrence remains. For treating moderate‐to‐severe colitis, oral molecular‐targeted drugs are a promising new option; however, their high cost and narrow target population make them difficult to apply in clinical practice. Therefore, identification of an effective and convenient treatment method remains a primary problem.

In recent years, plasma has received considerable attention for its applications in different fields, including wound healing, surface disinfection,^[^ ^27^ ^]^ blood coagulation, cancer treatment,^[^ ^28^ ^]^ and skin treatment.^[^ ^21^, ^29^ ^]^ PAS^[^ ^30^ ^]^ includes a variety of active oxygen and nitrogen species, but most of them are short‐acting particles that are finally dissolved in solution in the form of nitrate, nitrite particles, and hydrogen peroxide. Previous studies have found that PAS, mainly comprising active oxygen, has a certain killing effect on tumor cells^[^ ^31^ ^]^; some studies have also found that PAS has the potential to treat skin diseases like psoriasis.^[^ ^32^ ^]^ Further, PAS treatment can significantly improve the rate of wound healing and can be used to treat diabetic foot ulcer,^[^ ^33^ ^]^ which is a severe neurological wounds. Therefore, we aimed to explore the role of PAS in UC treatment. Promisingly, PAS treatment on a time gradient, especially PAS treatment for 2, 10, and 30 min, showed good therapeutic effects in a mouse model of colitis, mainly because it can effectively reduce the apoptosis of intestinal epithelial cells and promote mucosal repair. Further, the results revealed that PAS reduces the production of intestinal inflammatory factors and positively affects redox homeostasis.

Although PAS has been proven to play a positive role in the treatment of many diseases owing to its complex composition and the extremely short existence of most particles, the specific underlying mechanism remains unknown. Previous studies have found that adding sodium nitrite to diet is beneficial for colitis recovery in mice^[^ ^34^, ^35^, ^36^ ^]^; some studies have also found that administration of NO prodrugs can alleviate colitis,^[^ ^37^, ^38^ ^]^ suggesting an effective role for NO‐related pathways in colitis treatment. Based on these studies, we designed a Nitro‐free PAS‐generating device to produce PAS without nitrogen, and the results showed that the therapeutic effect of PAS was significantly weakened or even eliminated, indicating that nitrogen and its compounds are irreplaceable in the treatment of colitis using PAS.

In the past, most studies used differential photometry to analyze PAS components, which may have certain errors. Therefore, we used ion chromatography, which is more accurate, to standardize PAS. To exclude the influence of other ions on the results, we used deionized water instead of PBS or other buffers to prepare the PAS. We found that with the fixed equipment and preparation time, the NO_2_^−^ and NO_3_^−^ contents in the produced PAS were stable and could be effectively stored at a specific temperature.

Unlike previous studies,^[^ ^36^ ^]^ we found that PAS exerted obvious therapeutic effects when the content of NO_2_^−^ and NO_3_^−^ was very low. We hypothesized that although NO_2_^−^ and NO_3_^−^ are the main components that exert therapeutic effects, other active ingredients in PAS are required to maintain their active state. Previous studies have indicated that there is a large amount of NO in the microenvironment of individuals with colitis,^[^ ^39^ ^]^ and there is also an application to detect NO content for evaluating colitis activity. However, one study revealed that even in the presence of excessive NO in the microenvironment, the intestinal epithelial endogenous NO synthesis pathway is blocked. Excess exogenous NO is mainly produced by immune cells and cannot be utilized by intestinal epithelial cells.^[^ ^40^ ^]^ After the administration of NO precursor drugs, endogenous NO levels in the intestinal epithelium increase and promote mucosal proliferation and repair.^[^ ^40^ ^]^ This may provide insights into the therapeutic potential of PAS. After entering the intestinal tract, PAS forms an active nitrogen buffer pool. This can neutralize the exogenous NO from intestinal cells to form nitrogen oxides; thus, the product is a precursor that allows intestinal epithelial cells to produce endogenous NO and promote their repair. However, these views are only speculations and need to be further confirmed in our subsequent studies. Further, stable preparation of PAS makes the results of subsequent experiments more reliable and provides a good template for the clinical application of PAS in the future, which is one of the innovations in our research.

In recent years, the interaction between intestinal flora and diseases has become a research hotspot. Several studies have shown that disturbance of the gut microbiota is an important mechanism underlying the progression of UC.^[^ ^34^ ^]^ Several clinical trials have demonstrated that FMT can be effective in treating UC; however, the results remain controversial. When the intestinal epithelial mucosa is damaged, translocation of microbes occurs and disrupts the homeostasis of intestinal bacteria. The resulting toxic substances or metabolites play an important role in promoting colitis. Microbiota disorders can also be alleviated by repairing the intestinal mucosal barrier. Therefore, the interaction between the intestinal mucosal barrier integrity and intestinal bacteria is an important intervention target for colitis. Our results showed that PAS effectively inhibited inflammation and restored the colonic mucosal barrier in colitis treatment, mainly by significantly upregulating the expression of ZO1 and Occludin. Notably, PAS was first used for sterilization and purification. Several studies have demonstrated that it has a significant effect on bacteria. Some studies have used PAS atomizers to sterilize the air. Further, most intestinal bacteria rely on anaerobic respiration under hypoxic conditions and nitrogen oxides are one of the materials used for their survival. After nitrogen‐containing PAS enters the intestinal tract, whether it can change the microbiota by affecting the nitrogen levels in the microenvironment remains to be studied. Our results showed that the microbiota of mice fed with PAS, but not its metabolites, had a certain therapeutic effect; further, 16S sequencing revealed that after PAS treatment, the intestinal microbiota profile was significantly different from that of the DSS model mice. After PAS intervention, the α‐diversity of the intestinal flora increased, and the beneficial bacteria *Lactobacillus reuteri* could be recovered to a certain extent after PAS intervention. Additionally, PAS reduced the abundance of *Helicobacter typhlonius*, which was increased after DSS induction. In future, we will select appropriate probiotics to combine with PAS or with drugs targeting specific harmful bacteria to improve the efficacy of PAS.

Recurrence is a clinical characteristic of colitis and its reduction is one of the purposes of treatment. In previous experiments, we confirmed that PAS has the potential to treat colitis, which may be achieved by providing a nitrogen buffer pool and regulating the intestinal flora. After a period of PAS pre‐treatment, whether it influences DSS‐induced colitis with a certain buffering effect needs to be further explored. Therefore, we continued to establish a DSS‐induced colitis model to simulate clinical relapse. We found that after 2 weeks of PAS pre‐treatment followed by DSS intervention, the colonic injury and inflammation levels were significantly lower than those in the DSS direct intervention group, suggesting that PAS loading has a certain buffering effect. This suggests that the use of PAS maintenance therapy is beneficial to prevent colitis recurrence.

## 4 Conclusion

Based on these results, we found that 2 min treatment of PAS normalized using ion chromatography has the potential to treat colitis and prevent its recurrence. Specifically, PAS promotes intestinal mucosal repair, reduces inflammation, improves redox homeostasis, increases the abundance of the beneficial bacterium *Lactobacillus reuteri*, reduces the abundance of the harmful bacterium *Helicobacter typhonius*, and regulates intestinal bacterial homeostasis.

## 5 Experimental Section

### Production of Plasma‐Activated Solutions

The plasma generator that was used to produce plasma at room temperature is shown in Figure 1. CAP was produced in a helium‐containing atmosphere with a voltage of 12.4 kV and a flow rate of 15 L min^−1^. Every 6 mL solution was placed 3 cm below the flow of CAP at different treatment times, and then the solutions were tested via ion chromatography.

### DSS‐Induced Colitis Mouse Model

Five‐ to six‐week‐old specific pathogen‐free (SPF) female C57BL/6J mice were purchased, maintained with five animals per cage and housed in a standard SPF facility of Xi'an Jiaotong University with a 12 h light and 12 h dark cycle at 22 °C. Colitis was induced by administration of DSS (molecular mass 36–40 kDa, MP Biologicals, Solon, OH, USA) through drinking water every 2 days for 7 days.

### Treatments and Sample Collection

After 1 week of acclimation, mice were randomly divided into four groups, including the control group, DSS with PBS group, DSS with PAS group and control with PAS group. Animals were provided with free access to tap water supplemented with or without 2.5% DSS for 7 days, followed by free access to PBS or PAS for the following 2 weeks. Body weight was measured daily for the entire duration of the study. The disease activity index (DAI) was assessed daily to evaluate the severity of colitis. The mice were sacrificed after anesthesia, the length of the colon was measured, and fecal samples were collected sterilely from all mice and stored at −80 °C for future analysis. Plasma was obtained by centrifugation (3000 rpm for 15 min at 4 °C) and then stored at −80 °C. A 5 mm segment of the mid‐colon was flushed with PBS and then fixed in 10% formalin for subsequent histology analysis, while the remaining colon tissue was washed with PBS and snap‐frozen in liquid nitrogen for future analysis.

To study the prophylactic efficiency of PAS in the context of colitis, another experiment was conducted using five‐ to six‐week‐old SPF female C57BL/6J mice. After 1 week of acclimation, mice were randomly divided into four groups, including the control group, DSS group, PAS with DSS group and control with PAS group, with 5 mice per group, and received free access to PBS or PAS for 2 weeks, with or without 2.5% DSS in drinking water for the next 7 days to induce colitis. The method of collection was consistent with the previous description. Next, during the 2nd week, fresh feces (gut microbiota with or without its metabolites) were collected sterilely from the control group and PAS group for subsequent fecal microbiota transplantation (FMT) or sterile fecal filtrate transplantation (SFF). For the fecal transplants, fresh feces from each group were pooled, homogenized, and diluted in sterile saline to a final concentration of 50 mg feces/mL. Pooled samples were centrifuged at 100×g for 2 min, and the supernatant was filtered through 70 µm filters and then used for FMT treatment. For SFF, the supernatants were collected and passed through 70 and 0.22 µm filters. After 7 days of drinking water with 2.5% DSS, a total of 100 µL of the solutions used for FMT or SFF was administered orally per mouse daily for 2 weeks.

### Histological Analysis

For morphological measurements, formalin‐fixed colon tissues were sectioned and stained with hematoxylin and eosin. The extent of inflammatory infiltration, histopathological changes in crypt structure, ulceration, and crypt loss, the presence of ulcers, and the presence or absence of edema were evaluated, and the histological score was determined as previously described.^[^ ^41^ ^]^

### Terminal Deoxynucleotidyl Transferase‐Mediated dUTP Nick End Labeling (TUNEL) Assay

The formalin‐fixed colonic sections were also subjected to TUNEL staining using a TUNEL assay kit. Nuclei were stained, and images were acquired using a fluorescence microscope (Carl Zeiss AG, Jena, Germany). Hoechst 33342 and images were acquired using a fluorescence microscope (Carl Zeiss AG, Jena, Germany). The number of apoptotic cells was counted as described previously.^[^ ^42^ ^]^

### Confocal Immunofluorescence

Confocal immunofluorescence for ZO‐1 and Occludin expression in mouse colonic tissue was performed by standard methods. Colon cryosections were fixed in acetone. The cryosections were permeabilized with 0.1% Triton X‐100 (Sigma, X100) in PBS at room temperature for 5 min. The cryosections were then blocked in normal serum (Invitrogen, 50197Z) and labeled with primary antibodies in a blocking solution overnight at 4 °C. After PBS washes, the sections were incubated in Alexa Fluor‐488‐, Cy‐3‐, or Alexa Fluor‐647‐conjugated secondary antibodies (Invitrogen, A11078, A10521, and A21244). ProLong Gold antifade reagent (Invitrogen, P36931) containing DAPI as a nuclear stain was used to mount the sections on glass slides. The slides were examined using a Leica SP8 confocal fluorescence microscope. Images were processed with LAS X software (Leica Microsystems, Penn State College of Medicine).

### Transmission Electron Microscopy (TEM) Analysis

A 5 mm segment of fresh colon tissue was flushed with PBS and fixed in 2.5% glutaraldehyde at 4 °C for 4 h. After being rinsed in PBS, the tissue was further fixed in PBS containing 1% osmium tetroxide for 2 h at room temperature, rinsed in PBS, and dehydrated. The tissues were then embedded in Epon 812 overnight. Embedding was then performed in Epon 812, and curing was performed in an oven at 60 °C for 48 h. Sections of 80 nm thickness were cut on an ultramicrotome (RMC MTX) using a diamond knife. The sections were deposited on single‐hole grids coated with Formvar and carbon and double‐stained in aqueous solutions of 8% uranyl acetate for 25 min at 60 °C and lead citrate for 3 min at room temperature. Images were acquired by a transmission HT7700 electron microscope (Hitachi, Tokyo, Japan).

### Western Blotting

Total protein of colons were extracted in RIPA lysis buffer (Beyotime,P0013B) containing protease inhibitor and phosphatase inhibitor. Protein concentrations were determined with a BCA Protein Assay Kit after ultrasound at 4 °C. After gel electrophoresis, protein was transferred onto polyvinylidene fluoride membranes (Millipore,Billerica, MA,USA). The membranes were blocked by NcmBlot blocking buffer for 0.5 h (New Cell&Molecular Biotech,P30500), then incubated in the primary antibodies over night at 4 °C(ZO‐1 Polyclonal antibody,Proteintech,21773‐1‐AP;Occludin Polyclonal antibody,Proteintech,27260‐1‐AP). Following incubation with the HRP‐conjugated secondary antibody (Cell Signaling Technology) for 1 h at room temperature, an ECL kit (New Cell&Molecular Biotech;P2300) was used to visualize the immunocomplex.

### qRT‐PCR

Total RNA was isolated using the Total RNA Extraction Kit (Feijie,220 010). cDNA was synthesized from 1 µg of total RNA using the PrimeScript RT Master Mix (Perfect Real‐Time) (Takara,RR036A).Reactions were run on a CFX content instrument (Bio‐Rad) using 2×TSINGKE Master qPCR Mix(SYBR Green I) (TSE201).The primers used for RT‐qPCR are provided in Table S1 (Supporting Information).

### 16S rDNA Sequencing

QIAamp DNA Stool Mini Kit was used to extract DNA from fecal samples. The quality of DNA extraction was detected and evaluated by 1.2% agarose gel electrophoresis. The purified DNA was used as template, and the universal primers of 16S V3‐V4 region (357F 5′‐ACTCCTACGGRAGGCAGCAG‐3′; 806R 5′‐GACTACHVGGGTWTCTAAT‐3′) was amplified and the library was constructed. The constructed library was sequenced on the Illumina platform using Novaseq 6000 SP 500 Cycle Reagent Kit. The raw data obtained by sequencing were spliced and filtered to obtain clean data. Then cluster analysis was performed based on clean data to obtain operational taxonomic units (OTUs) at a similarity level of 97%. The species information was annotated by comparing the OUT representative sequence with the Silva 128 database. Based on taxonomic analysis, the composition of the samples at the level of Phylum, Class, Order, Family, Genus, and Species was clarified. α‐diversity analysis (observed OUTs index, Shannon index, Ace index, etc.) and β‐diversity analysis (Jaccard distance, Weighted Unifrac distance, etc.) were performed using mothur software (Version 1.33.3). Sequencing and quality control were done by HANBIO Co. Ltd.

### Statistical Analysis

Continuous variables are expressed as Mean ± SD unless otherwise specified. Sample size (n) for each statistical analysis was 5. The differences between two groups were assessed using a two‐tailed unpaired Student's *t* test (measurement data). Two‐way ANOVA was used to compare the means of three or more experimental groups. Statistical analyses were performed using GraphPad Prism 8.0 (GraphPad Software, San Diego, Canada). Statistical significance was defined as a P value of < 0.05. Differences were labeled as: ^*^ *p* < 0.05, ^**^ *p* < 0.01, ^***^ *p* < 0.001, ^****^ *p* < 0.0001, ns: no significant.

### Ethical Statement

Animal experiments were performed under a project license (No. 2021‐1523) granted by the Institutional Animal Care Committee of the Animal Experimental Center of Xi'an Jiaotong University, in compliance with the institutional guidelines for the care and use of animals.

## Conflict of Interest

The authors declare no conflict of interest.

## Supporting information

Supporting Information

## Data Availability

The data that support the findings of this study are available from the corresponding author upon reasonable request.
