# Supplementary material for: Plasma‐Activated Solutions Mitigates DSS‐Induced Colitis via Restoring Redox Homeostasis and Reversing Microbiota Dysbiosis
Source: Adv Sci (Weinh). 2023 Oct 23;10(34):2304044. doi: 10.1002/advs.202304044 (PMC10700679; doi:10.1002/advs.202304044)
Supplement: Supplementary file 1 — Supporting Information [file ADVS-10-2304044-s001.pdf]

ADVANCED SCIENCE
Open Access

Supporting Information

for *Adv. Sci.*, DOI 10.1002/advs.202304044

Plasma-Activated Solutions Mitigates DSS-Induced Colitis via Restoring Redox Homeostasis and Reversing Microbiota Dysbiosis

Tuanhe Sun, Kaijie Ren, Guimin Xu, Rulan Ma, Xueni Wang, Tianhao Min, Xin Xie, Anbang Sun, Yuyi Ma, Haonan Wang, Yong Zhang, Kun Zhu, Chengxue Dang, Guanjun Zhang and Hao Zhang**

Supporting Information

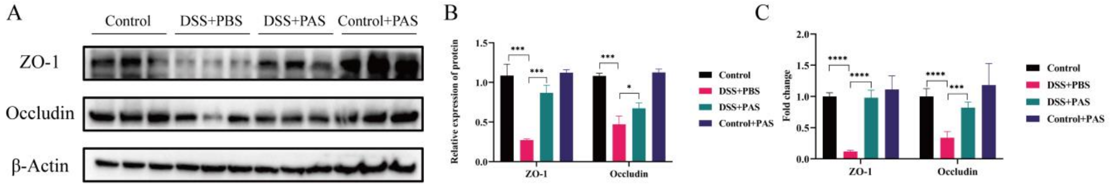

Figure S1 The expression of ZO-1 and Occludin of colons after treatment of PAS 2 min

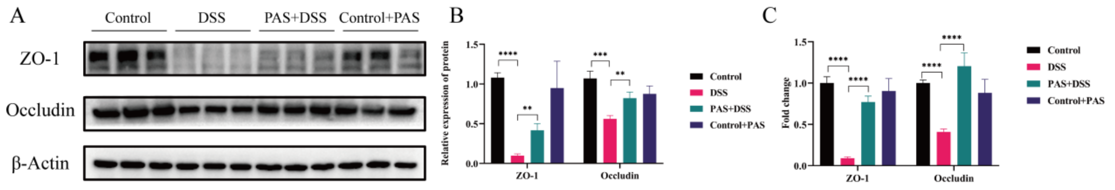

Figure S2 The expression of ZO-1 and Occludin of colons after pre-treatment of PAS 2 min

Table S1. Primers used for RT-qPCR

Genes	Primers	Sequence
ZO-1	Forward	GCCGCTAAGAGCACAGCAA
	Reverse	TCCCCACTCTGAAAATGAGGA
Occludin	Forward	TGAAAGTCCACCTCCTTACAGA
	Reverse	CCGGATAAAAAGAGTACGCTGG
Actin	Forward	GGCTGTATTCCCCTCCATCG
	Reverse	CCAGTTGGTAACAATGCCATGT
